# Combination of Chemically Characterized Pomegranate Extract and Hydrophilic Vitamins against Prolonged Fatigue: A Monocentric, Randomized, Double-Blind, Placebo-Controlled Clinical Trial

**DOI:** 10.3390/nu15132883

**Published:** 2023-06-26

**Authors:** Hammad Ullah, Eduardo Sommella, Alessandro Di Minno, Roberto Piccinocchi, Daniele Giuseppe Buccato, Lorenza Francesca De Lellis, Costanza Riccioni, Alessandra Baldi, Hesham R. El-Seedi, Shaden A. M. Khalifa, Gaetano Piccinocchi, Pietro Campiglia, Roberto Sacchi, Maria Daglia

**Affiliations:** 1 Department of Pharmacy, University of Napoli Federico II, Via D. Montesano 49, 80131 Naples, Italy; hammad.ullah@unina.it (H.U.); alessandro.diminno@unina.it (A.D.M.); d.buccato@studenti.unina.it (D.G.B.); lo.delellis2@libero.it (L.F.D.L.); alessandra.baldi.alimenti@gmail.com (A.B.); 2 Department of Pharmacy, University of Salerno, 84084 Fisciano, Italy; esommella@unisa.it (E.S.); pcampiglia@unisa.it (P.C.); 3 CEINGE-Biotecnologie Avanzate, Via Gaetano Salvatore 486, 80145 Naples, Italy; 4 Level 1 Medical Director Anaesthesia and Resuscitation A. U. O. Luigi Vanvitelli, Via Santa Maria di Costantinopoli, 80138 Naples, Italy; roberto.piccinocchi@policliniconapoli.it; 5 R&D Department, Esserre Pharma Srl, 00191 Rome, Italy; c.riccioni@esserrepharma.it; 6 International Research Center for Food Nutrition and Safety, Jiangsu University, Zhenjiang 212013, China; hesham@kth.se; 7 Department of Chemistry, Faculty of Science, Islamic University of Madinah, Madinah 42351, Saudi Arabia; 8 Psychiatry and Psychology Department, Capio Saint Göran’s Hospital, Sankt Göransplan 1, 112 19 Stockholm, Sweden; shaden.khalifa@regionstockholm.se; 9 Comegen S.c.S., Società Cooperativa Sociale di Medici di Medicina Generale, Viale Maria Bakunin 41, 80125 Naples, Italy; gpiccino@tin.it; 10 European Biomedical Research Institute of Salerno, Via De Renzi 50, 84125 Salerno, Italy; 11 Applied Statistic Unit, Department of Earth and Environmental Sciences, University of Pavia, Viale Taramelli 24, 27100 Pavia, Italy; roberto.sacchi@unipv.it

**Keywords:** prolonged fatigue, pomegranate extract, polyphenols, vitamin B complex, vitamin C

## Abstract

Prolonged fatigue is associated with non-pathological causes and lacks an established therapeutic approach. The current study is aimed at assessing the efficacy of a new food supplement (Improve™) based on a chemically characterized pomegranate extract and hydro-soluble vitamins (B complex and C). UHPLC-HRMS analysis of pomegranate extract showed the presence of 59 compounds, with gallotannins and ellagitannins being the most abundant phytochemicals. For the clinical study, 58 subjects were randomized into two groups, 1 and 2 (*n =* 29, each), which received either the food supplement or placebo. The effects of the food supplement against fatigue were assessed via validated questionnaires, recorded at time intervals t0 (at baseline), t1 (after 28 days), t2 (56 days), and t3 (after follow-up) in combination with the analysis of biochemical markers at t0 and t2. Fatigue severity scale (FSS) questionnaire scores were significantly decreased at the t2 and t3 time intervals in subjects treated with the food supplements, while the effect of the food supplement on a 12-Item Short Form Survey (SF-12) was not considerable. Moreover, the food supplement did not significantly affect biochemical parameters associated with fatigue and stress conditions. This study shows that the food supplement tested reduces prolonged fatigue following two months of supplementation in healthy subjects with mild prolonged fatigue.

## 1. Introduction

The word fatigue is derived from the Latin verb “fatigare”, which means “to tire”, and can be defined as a state of extreme tiredness brought about by physical or mental stress, resulting in a transient reduction of physical performance (also called physical fatigue) or mental performance (i.e., individual capacity to perform tasks requiring concentration or alertness, also called mental fatigue) [1,2,3]. Acute fatigue is a physiological reaction to intense and prolonged activity that is usually transient, does not interfere with routine activities, and maybe reduced with rest [4]. Prolonged fatigue occurs with the same symptoms as acute fatigue but with a longer duration (i.e., 30 days to 6 months) [5]. Chronic fatigue syndrome (CFS) or myalgic encephalomyelitis is best described as a profound disabling fatigue state (sense of exhaustion after activities and tiredness at rest), generally occurring in diseased subjects and lasting more than 6 months. It may interfere with daily tasks [6]. Epidemiological data indicate that the occurrence of prolonged or short-term fatigue in the general population is approximately 5–8%, while CFS figures are 3–4%. The Maastricht cohort study found that prolonged fatigue occurs in 21.9% of the adult working population and that this may also result in lower productivity at work [7]. Mood swings and lack of motivation and vitality are some of the effective consequences of fatigue [8]. Across studies, fatigue is also reported as one of the potential risk factors for the occurrence of accidents in occupational workers, where the possible role of fatigue is two-fold. First, it may reduce the capacity to process information regarding potential hazardous situations in the vicinity, and second, it may also alter the ability to respond adequately to hazardous situations as they unfold [9].

Despite the high occurrence rates of prolonged fatigue, there remains an absence of established and recommended pharmacological or non-pharmacological treatments. Some options have been explored, including exercise and cognitive behavioral therapy, but both show numerous limitations [10]. In terms of pharmacological treatments, certain drugs have been tested in patients with CFS due to serious pathological conditions where the need to reduce fatigue exceeds the adverse effects that these pharmacological treatments induce. Pemoline, amantadine, and modafinil have been applied in fatigue associated with multiple sclerosis, and donepezil and carnitine have been tested with advanced cancer, but weak and inconclusive evidence was observed regarding the efficacy of these substances. Pemoline and methylphenidate resulted in being effective in HIV-associated fatigue. Unfortunately, these results are based on only one study with a moderate number of participants [11].

*Punica granatum* L. (pomegranate) is an edible fruit tree species with a worldwide geographical distribution. It is well known for its high consumption and industrial value and for its nutritional and medicinal properties [12]. From the nutritional point of view, 100 g arils may provide about 72 kcal of energy, carbohydrate (16.6 g), protein (1.0 g), calcium (13 mg), sodium (1 mg), potassium (379 mg), magnesium (12 mg), copper (0.17 mg), iron (0.7 mg), vitamin C (7 mg), and niacin (0.3 mg) [12]. Moreover, the pomegranate fruit is rich in dietary polyphenols, including phenolic acids, flavonoids, and tannins [13,14]. The fruits could be considered functional foods because of the bioactive compounds they contain and their associated health benefits, including antioxidant, anti-inflammatory, cardioprotective, anticancer, hepatoprotective, antimicrobial, antiviral, antidiabetic, neuroprotective, and dermatologic effects [13]. In physically active subjects, pomegranate supplementation exerts beneficial effects improving endurance and performance after physical exercise through antioxidant and anti-inflammatory activities [15,16]. Moreover, a meta-analysis report of randomized controlled trials demonstrated an improvement in inflammatory markers (i.e., hs-CRP, TNF-α, and IL-6) in adults supplemented with pomegranate juice [17].

Hydro-soluble vitamins (Vitamin B complex and C) are a group of organic compounds that are required in trace amounts by the human body for nutritional, biochemical, and physiological purposes. The major sources of these vitamins are fruits, vegetables, legumes, cereals, meat, and eggs [18]. They are known to play an important role in the prevention of chronic degenerative disorders. The biological benefits attributed to these vitamins include antioxidant [19], anti-inflammatory [20], immunomodulatory [21], anticancer [21], cardioprotective [22], neuroprotective [23], metabolic regulating [24], and antianemic [25] effects. A literature review published by Werbach et al. [26] demonstrated the potential role of hydro-soluble vitamins and other nutrients in the clinical manifestation of chronic fatigue symptoms, as deficiencies in these were found across studies of CFS patients. In addition, Tardy et al. [8] reported that vitamin deficiency could be considered one of the possible causes of fatigue. A systemic review of the literature by Barnish et al. [27] demonstrated a protective role of nutrients, i.e., vitamins B, C, D, coenzyme Q10, L-carnitine, zinc, methionine, and nicotinamide adenine dinucleotide (NAD) in reducing fatigue symptoms both in healthy and diseased subjects.

Recently, Esposito et al. [5] established the efficacy, through validated questionnaires, of a food supplement consisting of the combination of a chemically characterized pomegranate extract and water-soluble vitamins against prolonged fatigue, in a one-month survey in which 78 subjects were recruited. The consumers reported a significant improvement in their condition without any unwanted effects.

On the basis of the above information, the aim of this monocentric, randomized, double-blind, placebo-controlled clinical trial was to assess the efficacy of a food supplement based on standardized chemically characterized pomegranate extract and hydro-soluble vitamins against prolonged fatigue by using validated questionnaires assessing fatigue level as a primary outcome of the trial. The secondary outcomes of the study were the improvement in the quality of life and fatigue-associated biomarkers of the enrolled subjects with mild to moderate prolonged fatigue.

## 2. Materials and Methods

### 2.1. Chemical Characterization of Pomegranate Extract with UHPLC-HRMS Analysis

Pomegranate extract was obtained by mixing a hydroalcoholic solution with different parts of the pomegranate fruit, particularly the peel, which includes the exocarp, mesocarp, and endocarp. The resulting solution was concentrated and spray-dried to obtain the standardized extract, which was chemically characterized by using a Thermo Ultimate RS 3000 coupled online to a Q-Exactive hybrid quadrupole Orbitrap mass spectrometer (Thermo Fisher Scientific, Bremen, Germany), equipped with a heated electrospray ionization probe (HESI II). For RP-UHPLC analysis, a Kinetex Biphenyl 100 mm × 2.1 mm, 2.6 µm (L × I.D, particle size, Phenomenex^®^, Bologna, Italy) column was employed at a flow rate of 0.4 mL/min. The mobile phases consisted of A) 0.1% CH_3_COOH in H_2_O and B) ACN plus 0.1% CH_3_COOH. Analysis was performed in gradient as follows: 0–30.0 min, 2–30% B; 30–38 min, 30–98% B; 99% B hold for 2 min; returning to initial conditions in 0.1 min. The column oven was set to 40 °C, and 5 µL of sample was injected. HRMS analysis was performed with Full MS (*m*/*z* 100–850) and data-dependent acquisition (dd-MS2 top N *=* 5). A resolution of 35.000 and 15,000 FWHM at *m*/*z* 200 was selected. Stepped normalized collision energy (NCE) values of 15, 25, and 30. Negative ESI- was employed. Source parameters were: Sheath gas pressure, 50 arbitrary units; auxiliary gas flow, 13 arbitrary units; spray voltage, −2.50 kV; capillary temperature, 260 °C; auxiliary gas heater temperature, 300 °C, S-lens RF value: 30 arbitrary units. Metabolite annotation was performed by comparison with in silico MS/MS Natural Product Library of MSDIAL v4.80 as previously reported [28].

### 2.2. Food Supplement Based on Pomegranate Extract and Hydro-Soluble Vitamins

Food supplements based on pomegranate extract, hydro-soluble vitamins (vitamins C and B complex), and placebo were produced by Esserre Pharma Srl (Rome, Italy) within European specifications for contaminants and microbiologic limits and notified to the Italian Health Ministry with the brand name Improve™ (notification number: I.5.i.h.2/2021/139191). The food supplement was produced in the form of soluble granulesin single-dose stick packs (1.6 g) containing 500 mg of pomegranate extract, 200 mg of vitamin C (L-ascorbic acid), 16 mg of niacin (nicotinamide), 7 mg of vitamin B2 (riboflavin), 6 mg of vitamin B5 (D-pantothenate, calcium), 5.55 mg of vitamin B1 (thiamine hydrochloride), 4 mg of vitamin B6 (pyridoxine hydrochloride), 200 µg of folate (pteroylmonoglutamic acid), 50 µg of D-biotin and 12.5 µg of vitamin B12 (cyanocobalamin). Placebo, in the same form of soluble granules, consisted of inert excipients (i.e., pomegranate flavor, beetroot red, maltodextrin, stevia, silicon dioxide, beta-carotene, caramelized sugar, citric acid, and xanthan gum), and considering the protocols of the double-blind study, it was made indistinguishable in color and flavor from the food supplement.

### 2.3. Clinical Trial Design

A monocentric, randomized, double-blind, placebo-controlled clinical trial was performed by COMEGEN—Società Cooperativa Sociale (Naples, Italy) to evaluate the efficacy of the food supplement based on pomegranate extract and vitamins C and B complex in adults with mild to moderate prolonged fatigue. The study was double-blind, both for the investigating physician and for the enrolled subjects. For this purpose, both the food supplement and the placebo were made to be unrecognizable in shape, weight, color, and, as far as possible, taste. The participants received verbal and written information regarding the study before signing a written consent. Protocol, letter of intent of volunteers, and synoptic documents regarding the study were approved by the Scientific Ethics Committee of A.S.L. Napoli 1 CENTRO (deliberation No. 1953—11 November 2022) and carried out in accordance with the Helsinki Declaration of 1964 (as revised in 2000). This study is listed on the ISRCTN registry (https://doi.org/10.1186/ISRCTN74944427; accessed on 14 April 2023).

The study design was composed of two experimental groups (*n =* 29, each group). The enrolled subjects were assigned to either of the two groups in a random and unpredictable way by means of simple randomization (allocation ratio 1:1). During the screening visit, the subjects were submitted to the Fatigue Severity Scale (FSS) questionnaire to understand if they met the requirements for participation in the study. During the first visit (t0), the recruited subjects consumed a stick-pack of the food supplement (group 1) or placebo (group 2). Then, at the baseline (t0), after 28 days (t1), and 56 days (t2), with a follow-up after a further 28 days (without treatment with placebo or food supplement), the recruited subjects were submitted to the completion of the FSS and 12-Item Short Form Survey (SF-12) questionnaires. Blood sampling at timed intervals measured biomarkers related to fatigue and stress conditions (i.e., at baseline (t0) and 56 days (t2) after the intake of the treatment or placebo).

Thus, the total duration of the study was about five months following the enrollment of the subjects and included two months of treatment.

### 2.4. Participants and Recruiting

Fifty-eight subjects aged 18–75 years of either sex were recruited by general practitioners through Comegen in February 2023 and randomized into two groups. Inclusion criteria for the study were defined as subjects with mild-to-moderate fatigue, with an FSS questionnaire score of less than 5, that were able to understand and sign the informed consent. Subjects with chronic fatigue (i.e., ˃six months) or an FSS score ≥ 5, pregnant or lactating women, subjects with cognitive impairments (that may hinder response to the questionnaires), subjects with a history of allergy to the ingredients used in the clinical trial (food supplement and placebo), subjects with chronic diseases or co-morbidities that may cause or aggravate fatigue symptoms (i.e., heart disorders, chronic hepatic, biliary and pancreatic diseases, neoplastic pathologies, rheumatological ailments, chronic hematological disorders, neuropsychiatric or neurological conditions, genetic-metabolic diseases, and diabetes type 1 or 2), candidates with malabsorption or eating disorders, subjects with a history of substance abuse or misuse (including drugs or alcohol), and subjects using multivitamin supplements within the previous month were excluded from the study. Additionally, subjects who used prescription or over-the-counter drugs that may have influenced cerebral excitability (i.e., tricyclic antidepressants, hypnotic drugs, anti-epileptics, antipsychotics, stimulants, antihistamines, muscle relaxants, dopaminergic drugs, and sedative-hypnotics) or drugs affecting systemic inflammation and levels of inflammatory mediators (i.e., non-steroidal and steroidal anti-inflammatory drugs) in the week prior to enrollment were rigorously evaluated for possible exclusion from the clinical study by the investigating clinician.

### 2.5. Outcome of the Study

The primary outcome of the present clinical study was to evaluate the efficacy of the food supplement consisting of pomegranate extract and hydro-soluble vitamins, to improve mild-to-moderate fatigue and persistent tiredness by the end of two months of treatment. The secondary outcomes of the study were to evaluate the effect of the food supplement on quality of life and any variations in the biomarkers related to fatigue and stress conditions.

### 2.6. Safety

Although no serious adverse events were expected related to the intake of the food supplement, the enrolled subjects were continuously monitored for the occurrence of any kind of adverse effects. Subjects with allergies to any of the ingredients of the food supplement were categorically excluded from the study.

### 2.7. Statistical Analysis

Sample size calculation was conducted using three 1-β power values (0.80, 0.95, and 0.99), a significance threshold value of α equal to 0.05, and three effect size values (Cohen’s f *=* 0.20, 0.25, and 0.30, respectively). The sample size was determined to be 58 participants (29 in each group) [29].

The effects of the treatments on the response variables (fatigue level, quality of life, and fatigue-associated biomarkers) were assessed through a linear mixed model (LMM), where the treatment (placebo and food supplement), the measurement times [i.e., at baseline (t0), after 28 days of treatment (t1) and after 56 days of treatment (t2)], and the age and sex of the subjects were entered into the model as fixed effects. The measurement × treatment interaction was also included among the independent variables. This interaction is the key variable for the primary endpoint, as it allows testing whether the trends for the placebo and food supplement treatments differ over the course of the measurement period. The identity of the subject was evaluated as a random effect, which provided control for differences between the enrolled subjects, regardless of fixed effects and their interactions. The same model was used for the analysis of the chemical biomarkers related to the secondary outcome. In this case, an independent LMM was run for each biomarker, where the fixed and random effects were the same as for the previous analyses. Analyses were performed using the lme4 [30] packages in R ver. 4.0.1 [31], and unless otherwise stated, data are reported as means ± standard errors.

## 3. Results

### 3.1. RP-UHPLC-HRMS Analysis of Pomegranate Extract

**Figure 1:**
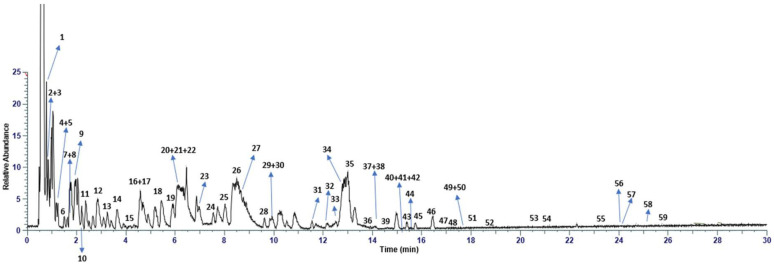
Chromatogram of pomegranate extract, with retention times on the *x*-axis and relative abundance on the *y*-axis. Each numbered peak represents one of the compounds identified in the pomegranate extract.

The pomegranate extract was chemically characterized by using an RP-UHPLC coupled with a Q Exactive hybrid quadrupole-Orbitrap mass spectrometer. By comparison with in silico MS/MS spectra, accurate mass, and molecular formula, 59 compounds were identified in the pomegranate extract, with confidence MSI lvl.2 [32], where the molecular formula and the corresponding mass are reported in Table 1 and Figure 1. The extract consists of gallotannins (galloyl-hexoside, HHDP-hexose, galloyl-hexoside_I, HHDP galloyl hexose, HHDP galloylhexose_I, HHDP galloylhexose_II, HHDP galloylhexose_III, hamamelitannin, HHDP galloylhexose_IV, HHDP galloylhexose_V, 1,3,6-tri-*O*-galloylhexose, 1,3,6-tri-*O*-galloylhexose_I, 1,3,6-tri-*O*-galloylhexose_II, 1,3,6-tri-*O*-galloylhexose_III, 1,3,6-tri-*O*-galloylhexose_IV, 1,3,6-tri-*O*-galloylhexose_V and 1,2,3,6-tetragalloylglucose), ellagitannins (pedunculagin, punicalagin, punicalagin_I, pedunculagin, punicalagin_II, pedunculagin, punicalagin, punicalin β, and punicalin β_I), hydroxybenzoic acids (gallic acid, protocatecuic acid, and ellagic acid), hydroxycinnamic acids (2-hydroxycinnamic acid, *p*-coumaric acid hexoside), flavanols (epigallocatechin), flavanones (naringenin, hesperidin, eriodictyol-7-*O*-hexoside, naringenin-7-*O*-hexoside), flavonols (myricetin, quercetin, kaempferol, myricetin-3-*O*-beta-L-galactopyranoside, quercetin-3-*O*-glucuronide, quercetin-3-*O*-rutinoside, quercetin-3-*O*-hexoside, quercetin-3-*O*-glucuronide, quercetin-3-arabinoside, kaempferol-7-*O*-hexoside, kaempferol-3-*O*-glucorhamnoside, quercetin-3-arabinoside_I, kaempferol-7-*O*-hexoside_I, kaempferol 3-alpha-L-arabinopyranoside, kaempferol-3-alpha-L-arabinopyranoside_I, Isorhamnetin, syringetin), flavones (apigenin, luteolin, apigenin-8-C-hexoside), dihydrochalcones (phloridzin), and miscellaneous compounds (citric acid and eryodictol). As expected, based on the relative intensity of MS data, hydrolysable tannins (i.e., gallotannins and ellagitannins) turned out to be the most abundant phytochemicals present in the extract.

### 3.2. Clinical Trial Design

**Figure 2:**
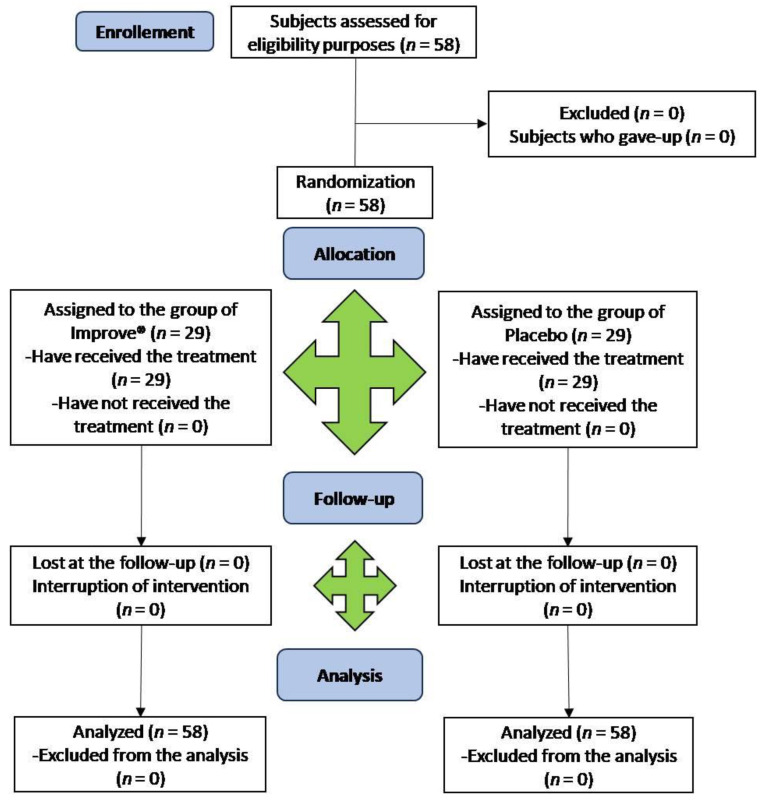
CONSORT flow diagram.

The study flow chart, produced in accordance with the CONSORT PRO reporting guidelines [33], is shown in Figure 2.

The two groups consisted of 58 subjects, including 22 males (corresponding to 38%, 12 of which were allocated to Group 1) and 36 females (corresponding to 62%, 17 of which were allocated to Group 1); both groups were administrated daily for 2 months, according to the parallel group design. Group 1 was initially treated with the placebo while Group 2 was treated with the food supplement. The participants from each group had similar sociodemographic characteristics and clinical data with no significant differences. The baseline characteristics of the subjects of each group are summarized in Table 2. Table 3 reports the data obtained from the answers of the questionnaires FSS and SF-12 at baseline (t0), after 28 days of the treatment (t1), after 58 days of the treatment (t2), and at the end of the follow up (t3).

**Figure 3:**
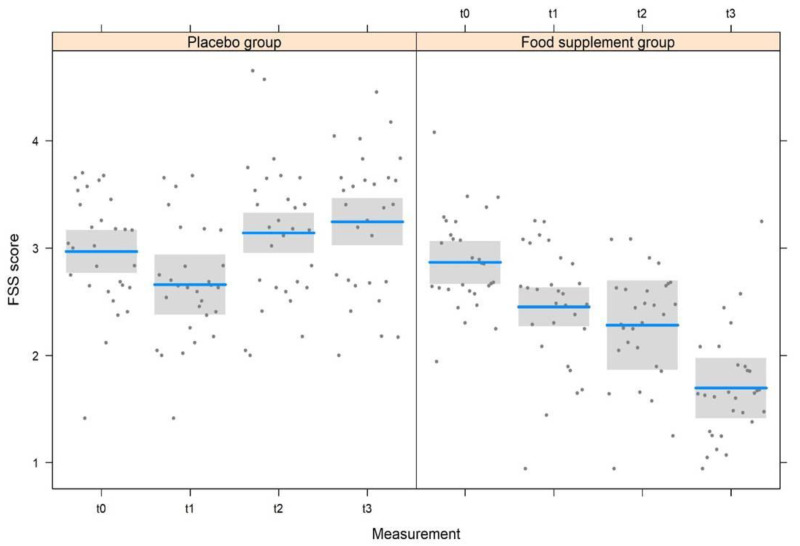
Variation of the FSS score at the four measurements in the two experimental groups.

**Figure 4:**
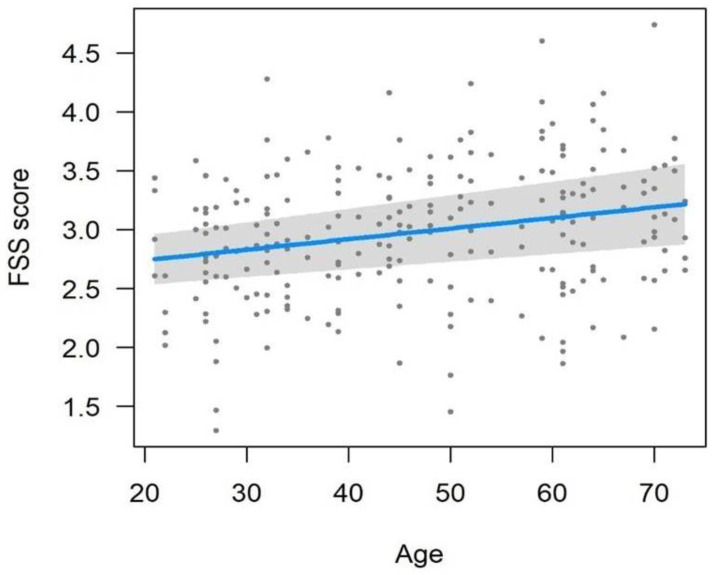
Variation of the FSS score in relation to the age of the subjects.

The LMM model used for the FSS questionnaire (Table 4 and Figure 3) identified highly significant effects (*p* < 0.001) for measurements, treatments, and also for their interaction. There was no significant effect based on gender, while a significant effect (*p =* 0.034) was identified related to the age of subjects. In accordance with FSS questionnaire scores, a statistically significant change was noticed (Table 3) from t0 to t1 (0.41 ± 0.16, t176 *=* 2.598, *p =* 0.010) and from t2 to t3 (0.59 ± 0.16, t176 *=* 3.680, *p* < 0.001). On the contrary, the decrease in FSS score from t1 to t2 (0.17 ± 0.16, t176 *=* 1.082, *p =* 0.28) was not significant. No significant differences were observed in the placebo group. Consequently, significant differences were noted between placebo and treated groups; however, it varies in accordance to the conditions, i.e., the FSS score between the two groups did not differ both at t0 (difference: 0.10 ± 0.17, t176 *=* 0.552, *p =* 0.58) and t1 (difference: 0.20 ± 0.18, t176 *=* 1.111, *p =* 0.27), but the FSS value of the treatment group was significantly lower than the placebo both at t2 (difference: 0.86 ± 0.18, t176 *=* 4.652, *p* < 0.001) and at t3 (difference: 1.55 ± 0.18, t176 *=* 8.379, *p* < 0.001). Finally, the FSS score increased significantly with the increasing age of subjects (Figure 4, 0.009 ± 0.004, t176 *=* 2.137, *p =* 0.034).

**Figure 5:**
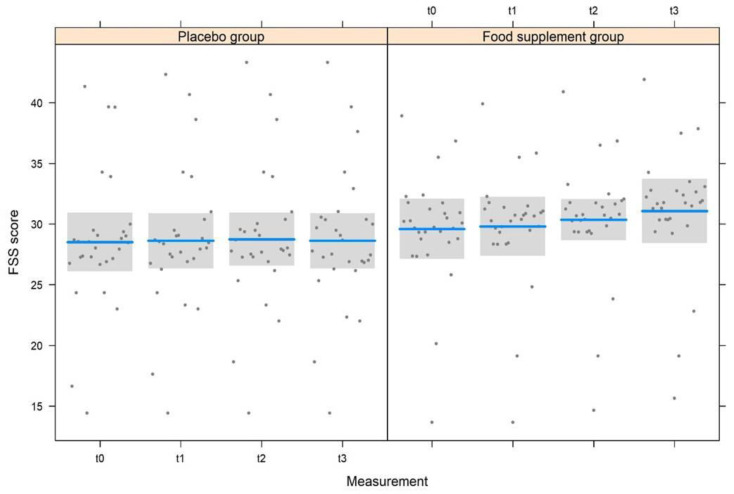
Variation of the SF-12 score across the four measurements in the two experimental groups.

Regarding the SF-12 questionnaire, the LMM model (Table 4) did not indicate any statistically significant effects. A slight tendency towards an increase in the questionnaire score was observed in the food supplement group (Figure 5), but this tendency was not statistically significant (*p =* 0.91).

Concerning the secondary outcomes, the LMM models applied to the biochemical markers did not show any differential effect of treatment on the response of the variables between the two measurements (t0 and t2, see Table 5). In fact, the measurement × treatment interaction was not significant in any case (Table 6). For cortisol, Mg^++^, K^+^, vitamin B12, folic acid, and vitamin D, no significant effect was observed for any independent variable at all. Significant differences between the initial value (t0) and final value (t2), regardless of the experimental treatment (i.e., placebo and food supplement), were noted for C-reactive protein (CRP), IL-6, Ca^++^, and Creatine phosphokinase (CPK) (Table 6). In particular, the values of CRP and IL-6 decreased significantly in both experimental groups between t0 and t2 (CRP: 0.88 ± 0.34, t80 *=* 2.607, *p =* 0.011; IL-6: 0.48 ± 0.24, t110 *=* 1.988, *p =* 0.050), while those of Ca^++^ and CPK increased between the two measurements (Ca^++^: 0.20 ± 0.09, t110 *=* 2.207, *p =* 0.029; CPK: 12.2 ± 6.1, t79 *=* 1.999, *p =* 0.049). Finally, the IL-6 value was significantly higher in the food supplement than in the placebo group, regardless of the measurement (0.48 ± 0.24, t110 *=* 1.980, *p =* 0.050).

Finally, during the three months of treatment, no subjects reported adverse reactions related to the administration of the food supplement, including the absence of allergies, and the principal investigator judged that this food supplement could be considered well tolerated.

## 4. Discussion

In this study, a combination of a chemically characterized pomegranate extract (rich in dietary polyphenols) and hydro-soluble vitamins (i.e., B complex and C) used as a food supplement was studied for its efficacy in the improvement in prolonged fatigue in a monocentric, randomized, double-blind, placebo-controlled clinical trial.

RP-UHPLC analysis coupled with a Q Exactive hybrid quadrupole-Orbitrap mass spectrometer showed the presence of 59 compounds in the pomegranate extract. The results support previous reports on the phytochemical composition of pomegranate extracts, although no study to date has determined the metabolic profile of these extracts in such detail [34,35,36,37].

According to European legislation, food supplements could be provided for the healthy population as they may exert nutritional or physiological effects but cannot exert therapeutic effects [38]. A large body of evidence suggests that prolonged fatigue is a non-pathological subjective condition. It is manifested by the persistent or repeated incidence of clinically unexplainable fatigue events (i.e., exhaustion and lack of energy, muscular pain, inability to focus, and orthostatic intolerance) following physical, mental, or infectious triggers, which may exert an impact on daily life routines [39]. Thus, subjects with mild to moderate prolonged fatigue with similar sociodemographic characteristics were recruited and monitored to test the potential effects of the food supplementation FSS score at four-time intervals (t0, t1, t2, and t3). At the first time point (t0), no statistically significant differences were found between FSS values recorded for the subjects enrolled in the placebo group or the food supplement group, showing that the recruited subjects demonstrated similar characteristics regardless of the group to which they belonged. At t1, the FSS scores were found to be lower than the values recorded at recruitment (t0) in both groups (placebo and treated groups), with no statistically significant difference between the two groups. However, at t2 and t3 following the intake of the food supplement, the FSS score decreased in a highly significant way in the treated group (*p* < 0.001), with values that dropped from 2.9 (t0) to 2.4 (t1), 2.3 (t2), and 1.7 (t3), showing an improvement in fatigue symptoms that continued even after the food supplement was discontinued. For the placebo group, the FSS scores at t2 and t3 increased and returned to the values recorded at t0, indicating that the placebo effect ends after a month (t1), and the subjects return to suffering from the same symptoms of prolonged fatigue recorded at baseline. Moreover, an independent effect was observed on the FSS score for the age of subjects, as there was a significant increase in the score with increasing age regardless of the treatment, showing the increased vulnerability of the individual to physical and mental fatigue with advanced age [40]. Based on this data, on average, the recruited subjects showed a good improvement in fatigue severity, with the intake of food supplements confirming our previous results [5].

As a secondary outcome, a questionnaire on the quality of life, SF-12, was used. The results show a slight, non-significant tendency toward an increase in the quality of life, with SF-12 values increasing from 30.5 (t0) to 32 (t3) for the food supplement group. For the placebo group, the recorded SF-12 values were constant (SF-12 values ranging from 27.6 at t0 to 27.7 at t3).

These results, taken together, are consistent with previous findings on pomegranate extract and hydro-soluble vitamins. Swamy et al. [41] reported on the anti-fatigue effects of pomegranate peel extract by assessing its efficacy on swimming performance in rats, which resulted in reduced malondialdehyde levels and increased glycogen contents. Moreover, acute ingestion of pomegranate extract 30 min before exercise delayed fatigue during exercise in a randomized, double-blind, placebo-controlled crossover study including 19 active individuals, indicating that pomegranate extract is ergogenic for intermittent running [42]. In addition, an inadequate intake of hydro-soluble vitamins is a challenging issue worldwide due to their rapid excretion from the body and the body’s subsequent inability to maintain them in long-term storage, thus increasing the risk of weakness, fatigue, apathy, and loss of appetite. An adequate intake of these vitamins may improve fatigue by targeting basic metabolic pathways supporting energy production, oxygen transport, mitochondrial function, and reduced free radical production [8]. Moreover, Vitamins B1, B5, B9, and C possess a beneficial role in maintaining brain structure, regulating intercellular connections, and biosynthesis of neurotransmitters, which ultimately results in improved psychological and cognitive functions [8]. The potential role of vitamin B complex in improving the symptoms associated with fatigue has been suggested across multiple studies. Supplementation of male athletes with B vitamins (B1 100 mg/tablet, B2 10 mg/tablet, B6 10 mg/tablet, B12 20 µg/tablet) significantly decreased the number of fatigue-associated complaints after exercise with an increase in blood glucose when supplemented with two tablets 3 days before exercise, and two tablets after exercise to support the recovery from fatigue [43]. Similarly, supplementation of obese adults with vitamin C (500 mg/day) for 4-weeks resulted in a decrease of general fatigue scores by 5.9 U as compared to control (1.9 U), with improvements in other parameters, such as heart rate and perceptions of exercise during moderate exercise [44]. Yeom et al. [45] demonstrated an improvement in fatigue symptoms, reflected by fatigue scores such as FSS and Visual Analogue Scale (VAS) along with biochemical parameters, i.e., blood vitamin C status, hemoglobin A1c, cortisol, aspartate aminotransferase, alanine aminotransferase, and CRP.

Regarding the other secondary outcomes (measured at t0 and t2), the intake of the food supplement did not show a significant effect on the biochemical markers between the beginning and the end of the clinical trial, as they remained quite constant and within normal ranges for both food supplement and placebo groups.

In particular, as far as IL-6 is concerned, the recruited subjects had IL-6 blood values typical of healthy subjects as defined by a recent meta-analysis, including 57 published studies with 3196 IL-6 values recorded in the blood of healthy donors, which reported that the average IL-6 blood levels range from 4.631 to 5.740 pg/mL in the majority of healthy individuals. Literature data reported that pomegranate polyphenols possess high biological activity during pathological inflammation. In fact, following an intake of pomegranate juice or extract, several clinical trials have shown a decrease in IL-6 blood levels in patients suffering from type 2 diabetes [46], obesity [47], non-alcoholic fatty liver disease [48], undergoing hemodialysis [49], or in athletes in which strenuous exercise may induce systemic inflammation [50]. However, the results reported in the literature and those of this study are only apparently discordant; in these aforementioned clinical trials, the treatment with pomegranate induced a reduction in elevated IL-6 blood concentrations caused by pathological inflammation, while in this present clinical trial, the IL-6 blood concentrations recorded in the recruited non-pathological subjects did not change before and after the food supplement treatment, remaining at normal uninflamed levels. It should be noted that in healthy subjects, IL-6 is synthesized by muscles and by adipocytes during their differentiation, without it being an indication of cellular damage or disease, and the presence of IL-6 is correlated to tissue regeneration, metabolism, bone homeostasis, and host defense [50]. Our results are similar to those published by other researchers, reported that serum markers of inflammation and muscle damage (i.e., IL-6, CRP, and CPK) did not show a significant effect between the beginning and the end of the clinical trial for the food supplement and the placebo groups [51,52]. In addition, our results agree with those obtained by O’Doherty et al. showing that supplementation with B vitamins and vitamin C, used alone or in combination, did not alter CRP concentrations in middle-aged, apparently healthy men [53].

This study shows strengths and limitations. The main strengths are that the efficacy of the food supplement was determined in a clinical trial with a robust design, suggesting that it is a safe and effective treatment for a large part of the population with mild to moderate prolonged fatigue. The main limitations are that it was not possible to show an improvement in the quality of life after the supplementation, probably due to the way the sample number calculation was conducted on the primary outcome, namely the FSS questionnaire, the number of subjects is insufficient to provide a statistically significant conclusion for this increase in SF-12, and the mechanism through which the food supplement exerts its effect is still largely unknown.

In conclusion, this study shows that a food supplement based on the combination of a chemically characterized pomegranate extract, B vitamins, and vitamin C, supplemented for two months of intake in healthy consumers, reduces prolonged fatigue. The mechanism of action is yet to be elucidated, but considering the high content of ellagitannins in this pomegranate extract that are able to reshape the gut microbiota by increasing eubiotic bacteria (*Lactobacillaceae*), likely through urolithins generated from the metabolism of polyphenolic components of pomegranate [54], and due to the suggested role of gut microbiota in prolonged and chronic fatigue [55], an in vitro study on the mechanism of action of this food supplement and pomegranate extract, evaluating the effect on the composition and functionality of gut microbiota, is currently in progress.

## Figures and Tables

**Table 1:** Identified compounds in pomegranate extract according to molecular formula, *m*/*z*, MS/MS, and intensity.

Peak	Tr	Compound	Molecular Formula	[M-H]-/[M-2H]2-	[MS/MS]	Error (ppm)	Intensity
1	0.65	Citric acid	C_6_H_8_O_7_	191.0194	111, 173	2.01	2.08 × 10^11^
2	0.92	Galloyl-hexoside	C_13_H_15_O_10_	331.0577	211, 169	1.54	1.23 × 10^8^
3	0.99	HHDP-hexose	C_20_H_18_O_14_	481.0693	300, 275	6.9	1.28 × 10^9^
4	1.08	Gallic acid	C_7_H_6_O_5_	169.0133	125	0.99	2.60 × 10^10^
5	1.16	Galloyl-hexoside_I	C_13_H_15_O_10_	331.0577	211, 169	1.52	2.29 × 10^7^
6	1.44	HHDP galloyl hexose	C_27_H_22_O_18_	633.0739	275, 300	0.49	3.78× 10^8^
7	1.77	Punicalin β	C_34_H_22_O_22_	781.0593	721, 601, 575, 392, 298, 273	0.1	6.04 × 10^8^
8	1.87	Protocatecuic acid	C_7_H_6_O_4_	153.0184	109	0.97	5.50 × 10^8^
9	1.98	Punicalin β_I	C_34_H_22_O_22_	781.0593	721, 601, 575, 392, 298, 273,	0.11	6.27 × 10^8^
10	2.38	HHDP galloylhexose_I	C_27_H_22_O_18_	633.0739	275, 300	0.5	7.92 × 10^9^
11	2.52	Epigallocatechin	C_15_H_14_O_7_	305.0673	261, 219, 179	0.58	1.54 × 10^8^
12	2.84	Pedunculagin (di-HHDP-hexose)	C_34_H_24_O_22_	783.0629	481, 300 275, 249	2.75	2.77 × 10^8^
13	3.24	HHDP galloylhexose_II	C_27_H_22_O_18_	633.0740	275, 300	0.47	5.38 × 10^9^
14	3.69	Punicalagin	C_48_H_28_O_30_	541.0262 **	301, 601, 275	2.49	8.36 × 10^7^
15	4.16	HHDP galloylhexose_III	C_27_H_22_O_18_	633.0738	275, 300	0.51	1.92 × 10^8^
16	4.57	Hamamelitannin	C_20_H_20_O_14_	483.0773	169, 271, 313	0.76	5.17 × 10^8^
17	4.59	Punicalagin_I	C_48_H_28_O_30_	541.0262 **	301, 601, 275	2.49	1.85 × 10^8^
18	5.44	Pedunculagin (di-HHDP-hexose)_I	C_34_H_24_O_22_	783.0629	481, 300 275, 249	2.75	2.77 × 10^8^
19	5.98	HHDP galloylhexose_IV	C_27_H_22_O_18_	633.0740	275, 300	0.47	2.22 × 10^9^
20	6.10	Punicalagin_II	C_48_H_28_O_30_	541.0266 **	301, 601, 275	2.49	2.44 × 10^8^
21	6.12	2-hydroxycinnamic acid	C_9_H_8_O_3_	163.0393	119	0.79	6.10 × 10^7^
22	6.15	*p*-Coumaric acid hexoside	C_15_H_18_O_8_	325.0929	163, 119	0.12	3.17 × 10^8^
23	6.96	Pedunculagin (di-HHDP-hexose)_II	C_34_H_24_O_22_	783.0629	481, 300 275, 249	2.75	2.27 × 10^8^
24	7.92	1,3,6-tri-*O*-galloylhexose	C_27_H_24_O_18_	635.0881	483, 465, 169	3.65	1.42 × 10^7^
25	8.14	Eriodictyol-7-*O*-hexoside	C_21_H_22_O_11_	449.1094	287, 259	2.47	2.99 × 10^8^
26	8.47	Punicalagin	C_48_H_28_O_30_	541.0266**	301, 601, 275	2.49	2.83 × 10^8^
27	8.77	HHDP galloylhexose_V	C_27_H_22_O_18_	633.0740	275, 300	0.47	4.13 × 10^8^
28	9.25	1,3,6-tri-*O*-galloylhexose_I	C_27_H_24_O_18_	635.0881	483, 465, 169	3.65	1.65 × 10^7^
29	9.62	1,3,6-tri-*O*-galloylhexose_II	C_27_H_24_O_18_	635.0880	483, 465, 169	3.63	6.02 × 10^6^
30	9.93	1,3,6-tri-*O*-galloylhexose_III	C_27_H_24_O_18_	635.0882	483, 465, 169	3.61	5.35 × 10^6^
31	11.22	1,3,6-tri-*O*-galloylhexose_IV	C_27_H_24_O_18_	635.0883	483, 465, 169	3.61	1.11 × 10^7^
32	11.56	Myricetin-3-*O*-beta-L-galactopyranoside	C_21_H_20_O_13_	479.0844	316	2.58	2.05 × 10^7^
33	11.72	1,3,6-tri-*O*-galloylhexose_V	C_27_H_24_O_18_	635.0883	483, 465, 169	3.61	2.19 × 10^7^
34	12.73	Quercetin-3-*O*-glucuronide	C_21_H_18_O_13_	477.0669	301	1.17	1.17 × 10^7^
35	13.01	Ellagic acid	C_14_H_6_O_8_	300.9987	229	3.75	6.98 × 10^8^
36	13.54	Quercetin-3-*O*-rutinoside	C_27_H_30_O_16_	609.1458	301, 463	3.52	4.34 × 10^7^
37	13.89	Naringenin-7-*O*-hexoside	C_21_H_22_O_10_	433.1143	271, 313	3.36	3.71 × 10^6^
38	13.91	Quercetin-3-*O*-hexoside	C_21_H_20_O_12_	463.0895	301	4.54	5.22 × 10^7^
39	14.59	Quercetin-3-*O*-glucuronide	C_21_H_18_O_13_	477.0669	301	1.17	1.08 × 10^7^
40	15.24	quercetin-3-arabinoside	C_20_H_18_O_11_	433.0780	301	3.95	5.47 × 10^7^
41	15.27	Eryodictol	C_15_H_12_O_6_	287.0568	259, 125	4.39	3.20 × 10^6^
42	15.38	Kaempferol-7-*O*-hexoside	C_21_H_20_O_11_	447.0943	285, 299	3.56	1.59 × 10^7^
43	15.40	Kaempferol-3-*O*-glucorhamnoside	C_27_H_30_O_15_	593.1523	285	3.27	1.09 × 10^8^
44	15.62	quercetin-3-arabinoside_I	C_20_H_18_O_11_	433.0780	301	3.95	3.86 × 10^5^
45	15.75	Kaempferol-7-*O*-hexoside_I	C_21_H_20_O_11_	447.0943	285, 299	3.56	9.14 × 10^7^
46	16.41	1,2,3,6-tetragalloylglucose	C_34_H_28_O_22_	787.1018	617, 465, 169	1.7	4.69 × 10^5^
47	16.63	Myricetin	C_15_H_10_O_8_	317.0309	178, 151	3.50	3.17 × 10^6^
48	16.99	Kaempferol-3-alpha-L-arabinopyranoside	C_20_H_18_O_10_	417.0833	284	2.88	1.11 × 10^7^
49	17.31	Apigenin-8-*C*-hexoside	C_21_H_20_O_10_	431.0996	269	3.58	3.52 × 10^7^
50	17.35	Kaempferol-3-alpha-L-arabinopyranoside_I	C_20_H_18_O_10_	417.0833	284	2.88	9.85 × 10^6^
51	17.57	Phloridzin	C_21_H_24_O_10_	435.1304	273, 167	3.64	1.67 × 10^7^
52	19.11	Hesperidin	C_28_H_34_O_15_	609.1816	301	3.11	5.37 × 10^6^
53	20.50	Quercetin	C_15_H_10_O_7_	301.0359	178, 151	1.29	1.03 × 10^7^
54	20.90	Kaempferol	C_15_H_10_O_6_	285.0402	151, 133	4.72	5.31 × 10^6^
55	23.58	Naringenin	C_15_H_12_O_5_	271.0613	177, 151, 119	5.01	1.38 × 10^6^
56	23.99	Apigenin	C_15_H_10_O_5_	269.0453	187, 119	3.5	1.65 × 10^6^
57	24.06	Luteolin	C_15_H_10_O_6_	285.0402	133, 151	4.83	5.57 × 10^6^
58	25.48	Isorhamnetin	C_16_H_12_O_7_	315.0517	300	4.25	1.48 × 10^6^
59	25.80	Syringetin	C_17_H_14_O_8_	345.0612	315, 330	3.87	4.72 × 10^6^

I *=* isomer; ** double charged.

**Table 2:** Demographic data of the study population at baseline (t0).

Characteristics of Enrolled Subjects	Group 1 (*n =* 29) Placebo	Group 2 (*n =* 29) Treated
Mean age (years):	49 ± 16	45 ± 15
○ Males (years)	46 ± 12	50 ± 16
○ Female (years)	44 ± 17	45 ± 16
Gender:		
○ Males	12	10
○ Females	17	19
Ethnicity:		
○ European	29	29

**Table 3:** Score values of the FSS and SF-12 questionnaires (mean ± SD and range of questionnaire values) for each measurement (t0, t1, t2, and t3) in the two experimental groups.

	Placebo				Treatment			
	t0	t1	t2	t3	t0	t1	t2	t3
FSS	3.0 ± 0.8	2.7 ± 0.9	3.1 ± 1.0	3.2 ± 0.8	2.9 ± 0.8	2.4 ± 0.9	2.3 ± 0.8	1.7 ± 0.7
	2–4	1–4	1–5	2–5	2–4	1–4	1–4	1–3
SF-12	27.6 ± 9.5	27.7 ± 9.7	27.8 ± 9.8	27.7 ± 9.8	30.5 ± 8.1	30.7 ± 8.1	31.2 ± 8.1	32 ± 8.2
	13–44	14–45	13–45	13–45	13–43	12–44	12–45	12–46

**Table 4:** Results of the LMM model for the score of the FSS and SF-12 questionnaires.

Model	Gdlnum	Gdl Den	F	*p*
FSS				
Measurement	3	176	6.104	<0.001
Treatment	1	206	30.39	<0.001
Gender	1	158	0.968	0.33
Age	1	194	4.568	0.034
Measurement × Treatment	3	176	17.73	<0.001
SF-12				
Measurement	3	179	0.219	0.88
Treatment	1	221	1.631	0.20
Gender	1	198	0.495	0.48
Age	1	217	1.983	0.16
Measurement × Treatment	3	179	0.171	0.92

**Table 5:** Values of biochemical markers (mean ± standard deviation, minimum and maximum) at the t0 and t2 measurements of the study in the two experimental groups.

Biochemical Markers	Placebo		Treatment	
	t0	t2	t0	t2
CRP (mg/L)	4.5 ± 1.8 (2–7)	3.1 ± 2.3 (0–7)	4.0 ± 1.7 (2–7)	3.6 ± 1.4 (2–6)
Cortisol (μg/dL)	15.2 ± 5.0 (7–25)	15.3 ± 5.6 (8–25)	17.3 ± 5.7 (8–25)	16.4 ± 6.2 (7–25)
IL-6 (pg/mL)	3.4 ± 1.4 (1–5.5)	2.9 ± 1.1 (1.3–4.8)	3.8 ± 1.4 (1.2–5.7)	3.4 ± 1.3 (1.1–5.1)
Mg^++^ (mEq/L)	2.0 ± 0.2 (1.7–2.2)	1.9 ± 0.2 (1.7–2.2)	1.9 ± 0.1 (1.7–2.2)	2.0 ± 0.1 (1.7–2.2)
K^+^ (mEq/L)	4.5 ± 0.4 (3.8–5.1)	4.5 ± 0.3 (3.8–5.1)	4.5 ± 0.4 (3.8–5.1)	4.4 ± 0.4 (3.8–5.1)
Ca^++^ (mEq/L)	9.2 ± 0.5 (8.6–10.2)	9.4 ± 0.5 (8.6–10.2)	9.3 ± 0.5 (8.7–10.3)	9.5 ± 0.5 (8.7–10.2)
CPK (U/L)	73.3 ± 34.5 (22–142)	90.7 ± 34.7 (31–145)	84.5 ± 33.8 (34–140)	91.6 ± 35.5 (21–143)
Vitamin B12 (pg/mL)	524.4 ± 189.4 (197–880)	559.6 ± 191.7 (242–869)	603.1 ± 188.6 (277–888)	583.4 ± 198.7 (192–840)
Folic acid (ng/mL)	15.8 ± 7.1 (4.1–26.3)	15.6 ± 7.0 (4.3–26.5)	15.5 ± 5.9 (4.3–25.3)	14 ± 6.9 (4–26.9)
Vitamin D (ng/mL)*	48.4 ± 24.9 (11–98)	48.2 ± 26.5 (12–92)	50.6 ± 26.4 (14–98)	60.6 ± 28.0 (13–96)

*expressed as 25-hydroxyvitamin D3.

**Table 6:** LMM model results for biochemical marker values.

Model	Dfnum	Df Den	F	*p*
CRP				
Measurement	1	110	6.796	0.011
Treatment	1	110	0.002	0.97
Gender	1	110	1.172	0.28
Age	1	110	0.072	0.79
Measurement × Treatment	1	110	2.511	0.12
Cortisol				
Measurement	1	80	0.130	0.72
Treatment	1	88	2.234	0.14
Gender	1	65	0.436	0.51
Age	1	83	0.049	0.82
Measurement × Treatment	1	80	0.210	0.65
IL-6				
Measurement	1	110	3.921	0.050
Treatment	1	110	3.775	0.050
Gender	1	110	3.112	0.08
Age	1	110	0.692	0.41
Measurement × Treatment	1	110	0.028	0.87
Mg^++^				
Measurement	1	59	0.505	0.48
Treatment	1	87	0.067	0.80
Gender	1	57	0.049	0.82
Age	1	79	0.013	0.91
Measurement × Treatment	1	59	2.825	0.10
K^+^				
Measurement	1	65	0.000	0.99
Treatment	1	83	0.093	0.76
Gender	1	54	0.956	0.33
Age	1	76	0.222	0.64
Measurement × Treatment	1	65	0.115	0.74
Ca^++^				
Measurement	1	110	4.873	0.029
Treatment	1	110	1.140	0.29
Gender	1	110	0.026	0.87
Age	1	110	0.601	0.44
Measurement × Treatment	1	110	0.078	0.78
CPK				
Measurement	1	79	3.998	0.049
Treatment	1	92	0.766	0.38
Gender	1	69	0.184	0.67
Age	1	87	3.413	0.07
Measurement × Treatment	1	79	0.695	0.41
Vitamin B12				
Measurement	1	110	0.048	0.83
Treatment	1	110	2.417	0.12
Gender	1	110	3.593	0.06
Age	1	110	0.068	0.79
Measurement × Treatment	1	110	0.600	0.44
Folic acid				
Measurement	1	110	0.461	0.50
Treatment	1	110	0.644	0.42
Gender	1	110	2.783	0.10
Age	1	110	0.196	0.66
Measurement × Treatment	1	110	0.271	0.60
Vitamin D				
Measurement	1	79	0.989	0.32
Treatment	1	83	2.350	0.13
Gender	1	60	1.093	0.30
Age	1	78	0.015	0.90
Measurement × Treatment	1	79	1.089	0.30

## Data Availability

Not available.
